# Safety of Granulocyte-Colony Stimulating Factor (G-CSF) administration for post-rehabilitation spinal cord injury patients: a phase I open-label study

**Published:** 2012-11

**Authors:** Hooshang Saberi, Nazi Derakhshan Rad, Mir Saeed Yekaninezhad, Keyvan Tayebi Meibodi, Hamide Tahmasebi

**Affiliations:** ^*a*^Department of Neurosurgery, Tehran University of Medical Sciences, Tehran, Iran.; ^*b*^Brain and Spinal Injuries Repair Research Center(BASIR) , Tehran University of Medical Sciences, Tehran, Iran.; ^*c*^Department of Epidemiology and Biostatistics, School of Public Health, Tehran University of Medical Sciences,Tehran, Iran.

## Abstract

**Background:**

Spinal cord injury (SCI) is a devastating neurological disorder causing various symptoms depending on the location, extent and degree of the damage. Granulocyte-colony stimulating factor (G-CSF) is a major growth factor in the activation and differentiation of granulocytes. This cytokine has been widely and safely employed clinically in different conditions over many years. On the other hand, the beneficial effects of G-CSF administration in spinal cord injury models have been shown in different studies.

**Methods:**

This study was conducted in the Brain and Spinal Injury Research Center and all of its procedures were approved by Tehran University of Medical Sciences Ethics Committee. A total of 11 patients with spinal cord injury were enrolled in the study and subcutaneous administration of GCSF was performed for 5 days in all of them. Follow up period was about 1 year. The American Spinal Injury Association (ASIA) scale used for motor and sensory assessment and the Spinal Cord Independence Measure (SCIM III) was used to evaluate the changes on the ability of performing basic everyday tasks.

**Results:**

Based on ASIA neurological examination scale, mean upper extremity motor score changed from 28.36 ± 19.64(Mean ± SD) to 29.91 ± 19.95 and there was no change in lower extremity motor score, light touch and pin prick sensory score had a modest improvement 3.91 and 1.27 scores respectively. However, changes in motor and sensory scores were statistically significant (P less than 0.05). The mean increase in SCIM total score was 3.11, modest improvements were also observed in the mobility in the room and self-care subscales (P less than 0.05).Mild side effects of GCSF treatment such as skin rash (1 case) and myalgia were observed. However, serious complications increasing the mortality and morbidity rates were not found.

**Conclusions:**

The findings of our study indicate that GCSF administration in the SCI patients appears safe and effective up to 1 year post injection. However, a randomized, placebo-controlled double blinded clinical trial with longer follow up is recommended to further assessed these findings.

**Keywords:**

Granulocyte colony stimulating factor, Treatment, Spinal cord injury, Rehabilitation


					Volume 4, Suppl. 1 Nov 2012
Publisher: Kermanshah University of Medical Sciences
URL: http://www.jivresearch.org
Copyright:
Congress of Iranian Neurosurgeons, 14-16 November 2012, Kermanshah, Iran
Paper No. 26
Safety of Granulocyte-Colony Stimulating Factor (G-CSF)
administration for post-rehabilitation spinal cord injury
patients: a phase I open-label study
Hooshang Saberi a,*
, Nazi Derakhshan Rad b
, Mir Saeed Yekaninezhad c
, Keyvan Tayebi Meibodi a
,
Hamide Tahmasebi a
a
Department of Neurosurgery, Tehran University of Medical Sciences, Tehran, Iran.
b
Brain and Spinal Injuries Repair Research Center(BASIR) , Tehran University of Medical Sciences, Tehran, Iran.
c
Department of Epidemiology and Biostatistics, School of Public Health, Tehran University of Medical Sciences,Tehran, Iran.
Abstract:
Background: Spinal cord injury (SCI) is a devastating neurological disorder causing various symptoms depending on
the location, extent and degree of the damage. Granulocyte-colony stimulating factor (G-CSF) is a major growth
factor in the activation and differentiation of granulocytes. This cytokine has been widely and safely employed
clinically in different conditions over many years. On the other hand, the beneficial effects of G-CSF administration in
spinal cord injury models have been shown in different studies.
Methods: This study was conducted in the Brain and Spinal Injury Research Center and all of its procedures were
approved by Tehran University of Medical Sciences Ethics Committee. A total of 11 patients with spinal cord injury
were enrolled in the study and subcutaneous administration of GCSF was performed for 5 days in all of them. Follow
up period was about 1 year. The American Spinal Injury Association (ASIA) scale used for motor and sensory
assessment and the Spinal Cord Independence Measure (SCIM III) was used to evaluate the changes on the ability of
performing basic everyday tasks.
Results: Based on ASIA neurological examination scale, mean upper extremity motor score changed from 28.36 
19.64(Mean  SD) to 29.91  19.95 and there was no change in lower extremity motor score, light touch and pin
prick sensory score had a modest improvement 3.91 and 1.27 scores respectively. However, changes in motor and
sensory scores were statistically significant (P less than 0.05). The mean increase in SCIM total score was 3.11,
modest improvements were also observed in the mobility in the room and self-care subscales (P less than 0.05).
Mild side effects of GCSF treatment such as skin rash (1 case) and myalgia were observed. However, serious
complications increasing the mortality and morbidity rates were not found.
Conclusions: The findings of our study indicate that GCSF administration in the SCI patients appears safe and
effective up to 1 year post injection. However, a randomized, placebo-controlled double blinded clinical trial with
longer follow up is recommended to further assessed these findings.
Key words:
Granulocyte colony stimulating factor, Treatment, Spinal cord injury, Rehabilitation
* Corresponding Author at:
Hooshang Saberi: Department of Neurosurgery, Tehran University of Medical Sciences, Tehran, Iran, Tel: 09121085771,
Email: hgsaberi@yahoo.com, (Saberi H.).

				

